# Lipoxin A4 analogue, BML-111, reduces platelet activation and protects from thrombosis

**DOI:** 10.1186/s12959-024-00606-7

**Published:** 2024-04-23

**Authors:** Shatha AlOmar, Joanne L Mitchell, Eman AlZahrani

**Affiliations:** 1 Department of Clinical Laboratory Sciences, King Saud University, Prince Turki Ibn Abdulaziz Al Awwal Rd, 12371 Riyadh, Saudi Arabia; 2 Cardiovascular Sciences, University of Birmingham, Birmingham, UK; 3 School of Pharmacy, University of Reading, Reading, UK

**Keywords:** Formyl peptide receptor 2/ALX, BML-111, Platelets, Thrombosis

## Abstract

Formyl peptide receptors (FPRs) are members of seven transmembrane G protein-coupled receptors superfamily that exhibit different responses based on the nature of stimulating ligand type. FPRs have been shown to be present in platelets and regulate their function. However, the effect of formyl peptide receptor 2 (FPR2/ALX) lipid ligands on platelets has not yet been addressed. Hence, we sought to study the role of FPR2/ALX ligand and lipoxin A4 lipid analogue, BML-111, in the modulation of platelet function and thrombus formation. Immunofluorescence microscopy showed subcellular distribution and peripheral mobilisation of FPR2/ALX in stimulated platelets. This variation in distribution was further confirmed using flow cytometry. BML-111 inhibited a range of platelet activities in a dose-dependent manner in response to several platelet agonists. This included aggregation, fibrinogen binding to integrin αIIbβ3, α-granule secretion, dense granule secretion, Ca^2 +^ mobilisation and integrin αIIbβ3-mediated outside-in signaling. The selectivity of BML-111 for FPR2/ALX was confirmed using FPR2/ALX deficient mice in flow cytometry assays. In *vitro* thrombus formation was also inhibited by various concentrations of BML-111. Moreover, the levels of vasodilator stimulated phosphorylation (VASP-S157) increased significantly after BML-111 treatment in resting and stimulated platelets via protein kinase A (PKA) independently of cyclic adenosine monophosphate (cAMP) signaling. Together, our findings demonstrate the significance of BML-111 as a modulator of platelet function via FPR2/ALX and unravel the thrombo-protective potentials of BML-111 induced signaling against thrombo-inflammatory diseases.

**Supplementary Information:**

The online version contains supplementary material available at 10.1186/s12959-024-00606-7.

## Key points

• Lipoxin A4 analogue, BML-111 reduces platelet function.
• BML-111 augments platelet inhibitory signaling pathway.

**Supplementary Information:**

The online version contains supplementary material available at 10.1186/s12959-024-00606-7.

## Introduction

The FPRs family is known for the structural variety of their ligands. This repertoire includes several ligands with diverse chemical properties and origins from natural peptides to synthetic non-peptide molecules [1, 2]. Among these ligands is LXA4, an eicosanoid that has been shown to play a vital physiological role in the resolution of inflammation [3, 4]. Interestingly, FPR2/ALX mediated signaling exhibits dual effects of pro-inflammatory and anti-inflammatory responses based on the stimulating ligand type. FPR2/ALX recognises both peptide, protein and lipid ligands, where peptide agonists mostly stimulate pro-inflammatory signaling events including the phosphorylation of extracellular signal-regulated kinases 1/2 (ERK1/2), Ca ^+ 2^ mobilisation, and superoxide generation [1, 5–7]. Interestingly, the presence of FPRs was reported in megakaryocyte and human platelets at transcript and protein levels [8, 9]. These findings raised growing research interests in the role of FPRs and their corresponding ligands in the regulation of thrombosis and haemostasis [10, 11].

Using a variety of independent methods, we demonstrate the subcellular distribution of FPR2/ALX in human platelets and the mobilisation of these receptors in response to stimulation. We also validated the selectivity of the LXA4 analogue, BML-111 towards FPR2/ALX in human and mouse platelets. A wide range of platelet function was reduced upon BML-111 treatment. Our data reveal that platelet response to stimulation was negatively impacted in the presence of BML-111 via modulation of PKA activation independently of cAMP.

## Methods

All the experimental work in the present paper was performed according to the protocols described in the supplementary material. This includes washed platelet preparation, immunofluorescence, immunoblotting, and several platelet functional assays such as aggregation, fibrinogen binding, P-selectin exposure, dense granule secretion, platelet spreading, clot retraction, calcium mobilisation, and thrombus formation.

The statistical significance for studies with more than 2 groups comparison was assessed using one-way analysis of variance (ANOVA). The in vitro thrombus formation assay was analysed using two-way ANOVA. Data presented as mean ± SEM and a P-value of ≤ 0.05 was considered to be statistically significant. Statistical analysis was performed using GraphPad Prism software (GraphPad, San Diego, CA, version 8.00).

## Results

### Distribution of FPR2/ALX in platelets

**Fig. 1:**
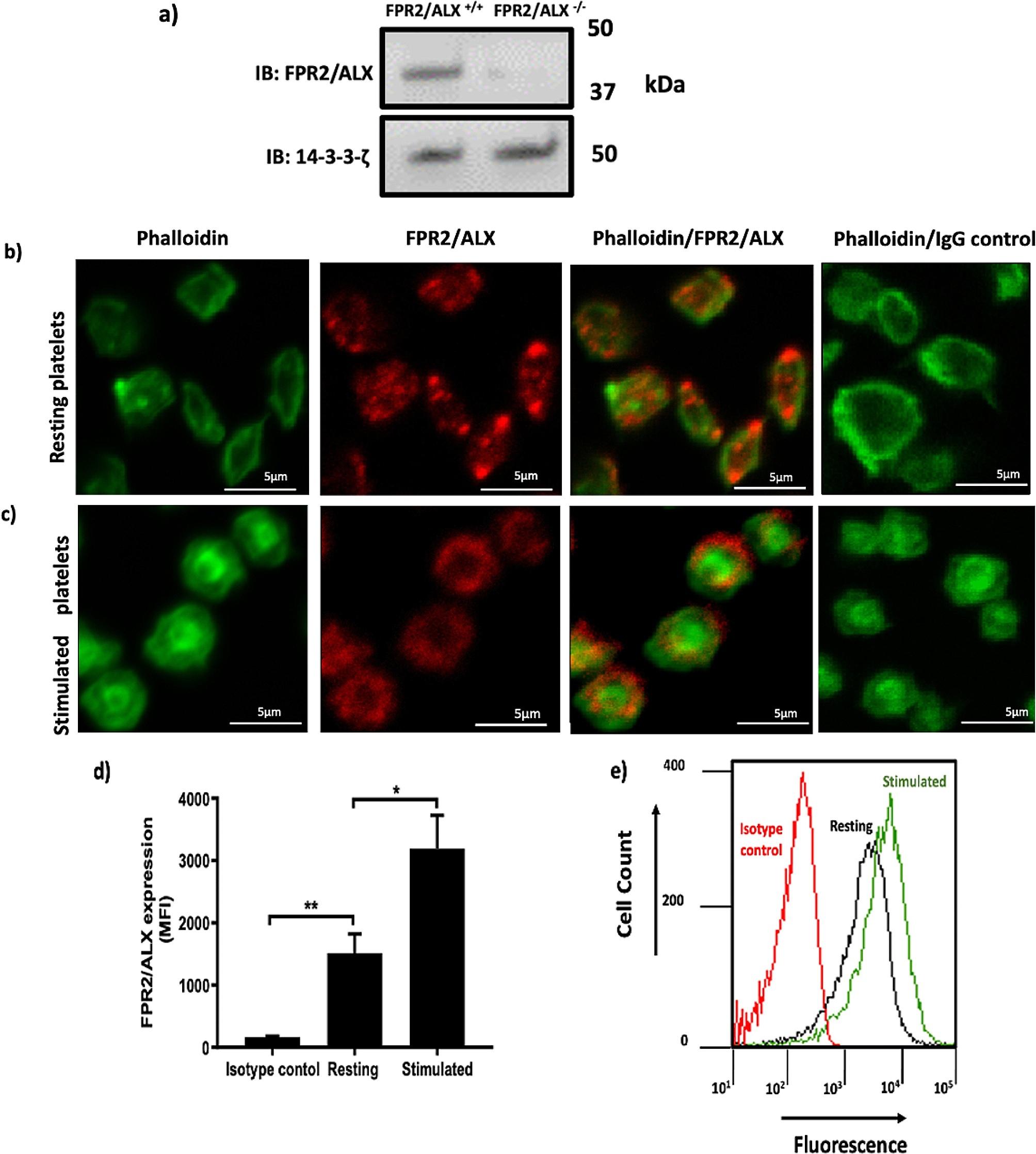
Immunofluorescence and flow cytometry analyses of resting and stimulated platelets confirm the presence of FPR2/ALX. (**a**) FPR2/ALX^+/+^ and FPR2/ALX^−/−^ mouse platelet were lysed and analysed by SDS-PAGE. Following blotting of the separated proteins on a PVDF membrane, the primary (rabbit polyclonal anti-FPR2/ALX antibody) and secondary (Cy5™ anti-rabbit IgG antibody) antibodies were used to detect FPR2/ALX. 14-3-3-ζ was included as a loading control. The membrane was scanned using a Typhoon 9400 Variable Mode Imager (GE Healthcare, UK) to visualise the bands. (**b**) Resting and (**c**) 5 µM U46619-stimulated platelets were fixed and permeabilised by using 0.2% (V/V) TritonTM x-100 before the addition of anti-FPR2/ALX antibody and Alexa Fluor 488-conjugated Phalloidin. Alexa Fluor 647-labelled anti-rabbit IgG antibodies were added to detect FPR2/ALX. As controls, Alexa Fluor 647-anti-rabbit IgG and Phalloidin were added. Negative controls excluded the non-specific binding. Platelets were then visualised by confocal microscopy, objective (1000x). The images shown are representative of several images taken for three separate donors. Human PRP [resting or CRP-XL (0.5 µg/ml) stimulated] was incubated with anti-FPR2/ALX antibodies and detected using Cy5TM anti-rabbit IgG secondary antibodies and analysed by flow cytometry. (**d**) The bar chart represents the surface expression of FPR2/ALX in platelets, and its expression increases compared to isotype control. (**e**) A representative histogram showing the elevation of FPR2/ALX levels in activated platelets (green) compared to resting platelets (black). The red line represents the isotype control. Data represent the mean ± SEM (*n* = 3) of the median fluorescence intensity values. *P ≤ 0.05 and **P ≤ 0.01 values were as calculated by One-way ANOVA

The presence of FPR2/ALX mRNA in megakaryocytes highlighted the expression of this receptor in human and mouse platelets [8, 12]. In addition, the expression of FPR2/ALX in human and mouse platelets has been previously confirmed at protein levels through immunoblot analysis [8, 13], however, the distribution of the receptor in human platelets has not been addressed yet. Therefore, we investigated the subcellular localisation of the receptor in permeabilised-resting platelets with the aid of anti-FPR2/ALX antibody. The specificity of the antibody was confirmed on FPR2/ALX-deficient platelet as shown in Fig. 1a. Using immunofluorescence microscopy, FPR2/ALX was observed around the periphery of platelets in resting condition. Interestingly, FPR2/ALX was also seen inside the cytosol of platelets (Fig. 1b). However, upon activation, FPR2/ALX seems to get mobilised towards the platelet periphery (Fig. 1c ). This finding was further confirmed using flow cytometry. Human PRP was stimulated with vehicle control or 0.5 µg/ml CRP-XL prior to the addition of anti-FPR2/ALX antibody and incubation for another 10 min. The secondary antibody (Cy5™ conjugated anti-rabbit IgG) was added and incubated for a further 10 min. Samples were then fixed with 0.2% (v/v) formyl saline, and the level of fluorescence was measured using a flow cytometer. In agreement with the imaging findings, variation in the distribution of FPR2/ALX receptors in resting and stimulated platelets was also noted (Fig. 1d, e).

### BML-111 inhibits platelet aggregation

**Fig. 2:**
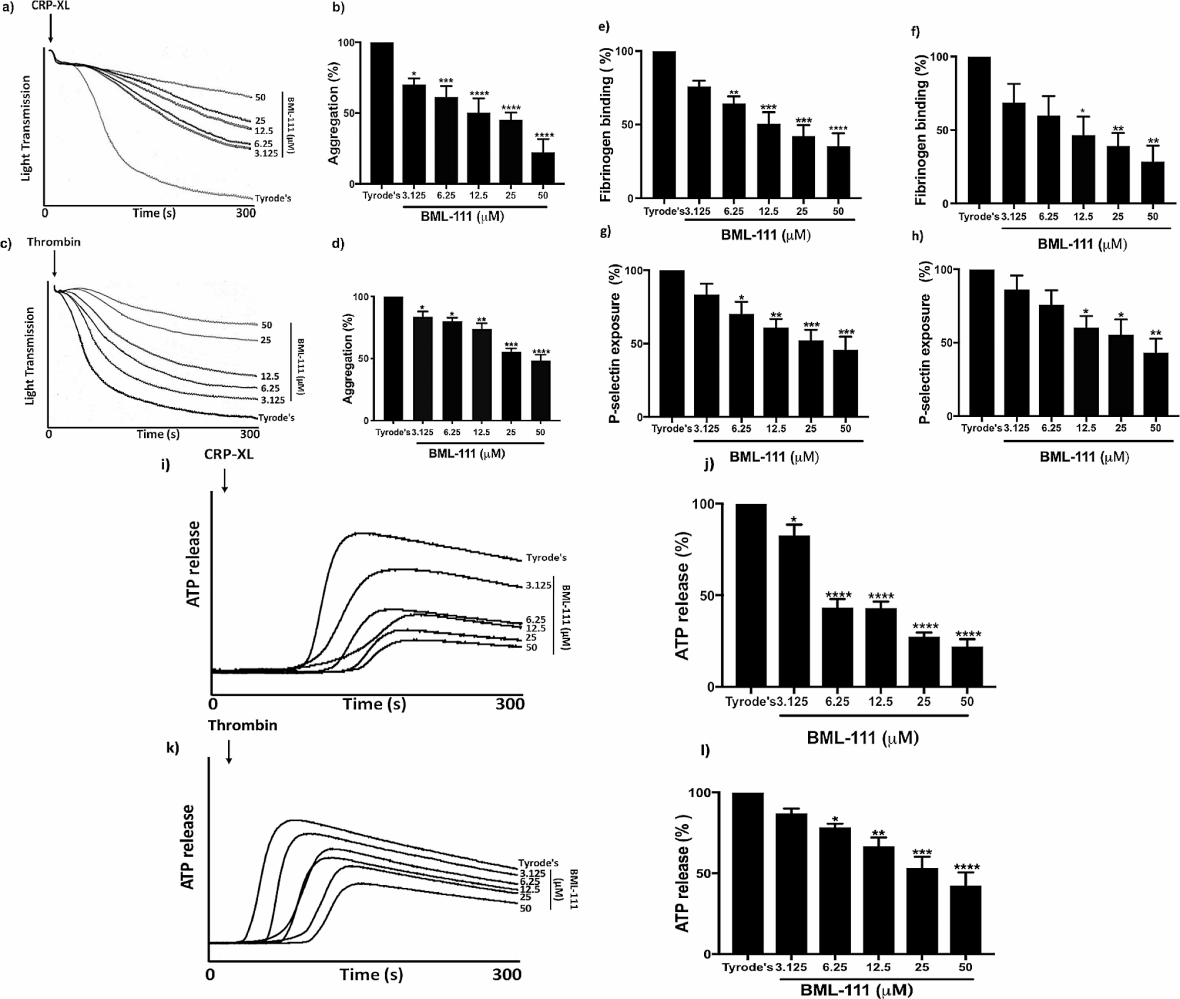
BML-111 inhibits platelet aggregation, integrin αIIbβ3 activation and secretion. Human isolated platelets (4 × 10^8^ cells/ml) were incubated with modified-Tyrode’s HEPES buffer or different concentrations of BML-111 (3.125, 6.25, 12.5, 25, and 50 µM) for 5 min and then stimulated with CRP-XL (0.25 µg/ml) and thrombin (0.05 U/ml). The change in light transmission was monitored and recorded for 300 s. (**a**, **c**) Representative aggregation traces from three separate donors. (**b**, **d**) Cumulative data from three different donors. The percentage of aggregation measured at 300 s with a vehicle control was taken as 100%. Human PRP was incubated with different concentrations of BML-111 (3.125, 6.25, 12.5 and 50 µM) or vehicle control (modified-Tyrode’s HEPES buffer) for 5 min prior to stimulating with (**e**) CRP-XL (0.25 µg/ml) or (**f**) thrombin (0.05U/ml) for 20 min followed by flow cytometry analysis. Integrin αIIbβ3 activation (inside-out signaling) was determined by measuring the level of fibrinogen binding to the platelets. The bar charts represent the percentage of fibrinogen binding compared with the positive control, which is defined as 100%. The level of P-selection exposure on the platelet surface was measured using anti-human CD62P antibodies by flow cytometry. The bar charts represent the percentage of P-selectin exposure compared with the positive control, which is defined as 100% (**g**, **h**). The level of ATP release was observed by lumi-aggregometery using a luciferin-luciferase detection system. (**i**, **k**) Representative traces for ATP release. (**j**, **l**) ATP secretion was calculated as a percentage of the area under the curve, where 100% was expressed as the level of ATP release achieved with a vehicle control at 300 s. Data represent mean ± SEM (*n* = 3). *P ≤ 0.05, **P ≤ 0.01, ***P ≤ 0.001 and ****P ≤ 0.0001 values were as calculated by One-way ANOVA.

BML-111 is a LXA4 analogue that has been proven to selectively bind FPR2/ALX receptors in platelet with the aid of FPR2/ALX-deficient platelets (supplementary Fig. 1a &b). We studied the impact of BML-111 on platelet aggregation following stimulation with crosslinked collagen-related peptide (CRP-XL), a selective GPVI agonist, and thrombin, an agonist for protease-activated receptors (PARs). Human isolated platelets were incubated with either vehicle control (modified-Tyrode’s HEPES buffer) or a range of different concentrations of BML-111 for 5 min prior to the addition of CRP-XL or thrombin, the tested BML concentrations were chosen based on a toxicity assay (supplementary Fig. 1c). The aggregation was monitored under stirring conditions at 37 °C for 300 s using light transmission aggregometry (Fig. 2). As shown in Fig. 2a and b, pre-treatment with BML-111 inhibited platelet aggregation in a dose-dependent manner when compared to the vehicle-treated sample in response to CRP-XL stimulation. Similarly, thrombin-induced platelet aggregation was also inhibited in the presence of increasing concentrations of BML-111 (Fig. 2c and d).

### BML-111 reduces inside-out signaling to integrin αIIbβ3 and platelet degranulation

Crosslinking of platelets occurs through the binding of fibrinogen to activated integrin αIIbβ3 that leads to the formation of platelet aggregates. Given the inhibitory effects of BML-111 on platelet aggregation, we sought to investigate the influence of this ligand on integrin αIIbβ3 activation using FITC-conjugated rabbit anti-human fibrinogen antibody via flow cytometry. Fibrinogen binding to CRP-XL stimulated platelets was reduced by around 35%, 49%, 57% and 64% at 6.25, 12.5, 25 and 50 µM of BML-111, respectively (Fig. 2e). The inhibitory effect of BML-111 was also observed in thrombin stimulated platelets at higher concentrations of BML-111 (Fig. 2f). These findings are in alignment with inhibited platelet aggregation upon stimulation with CRP-XL or thrombin following ligation of FPR2/ALX with BML-111. In addition, the impact of BML-111 on the α-granule secretion of platelets was analysed using PE-Cy5 conjugated mouse anti-human CD62P antibody by flow cytometry. As shown in Fig. 2, significant and dose-dependent inhibition of P-selectin exposure was observed when different concentrations of BML-111 were added upon stimulation with CRP- XL (Fig. 2g). Moreover, thrombin mediated exposure of P-selectin was significantly reduced when platelets were treated with BML-111 (Fig. 2h). Also, we measured the effect of BML-111 on ATP release from activated platelets using a luciferin-luciferase reagent. BML-111 treatment negatively impacted ATP release at various concentrations when stimulated with CRP-XL as demonstrated in Fig. 2i &j. Likewise, inhibition was also observed in thrombin stimulated platelets when compared to vehicle-treated samples as shown in Fig. 2k &l.

### BML-111 negatively impacts integrin αIIbβ3 outside-in signaling

**Fig. 3:**
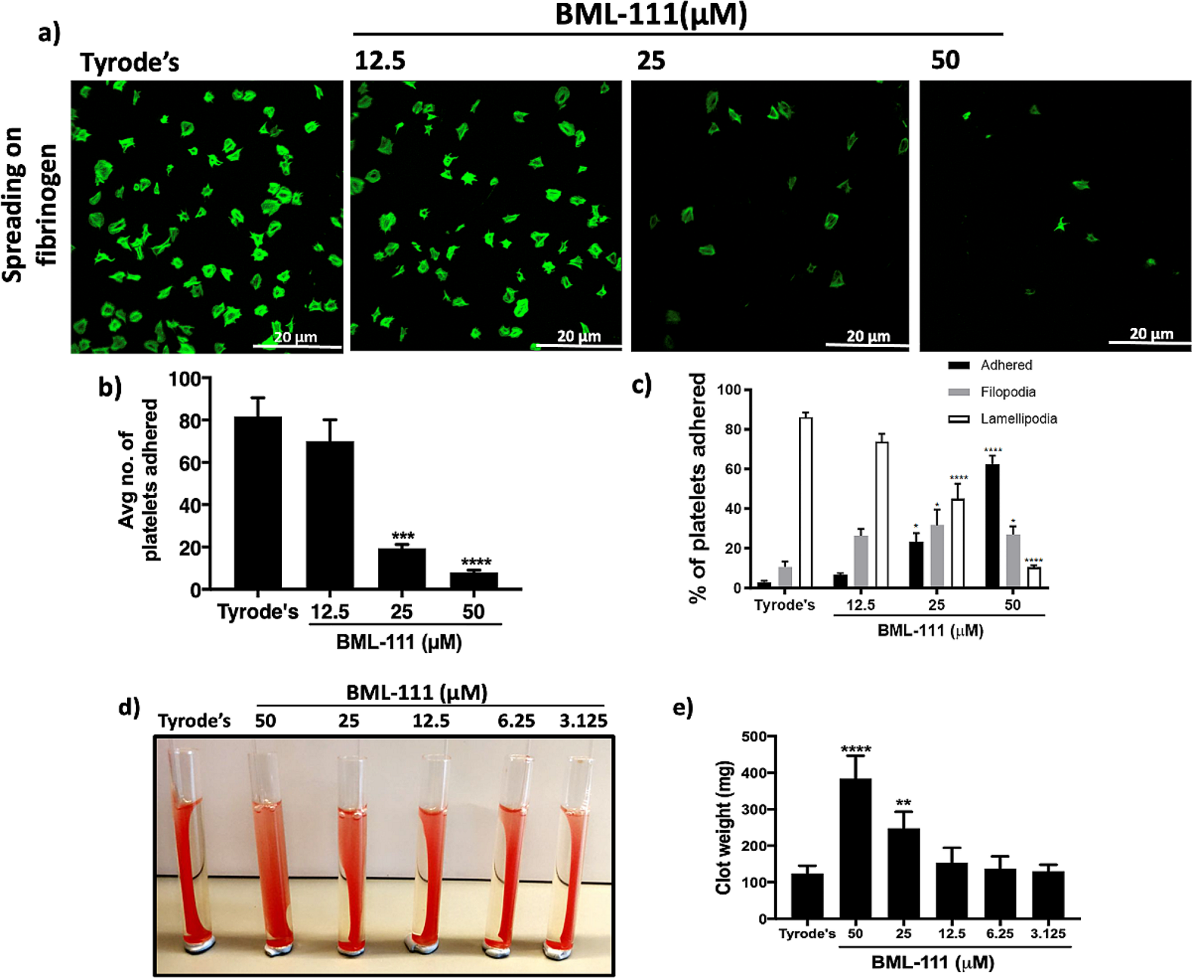
BML-111 prevents adhesion and spreading of platelets on immobilised fibrinogen and clot retraction. Human isolated platelets (2 × 10^7^ cells/ml) were incubated with modified-Tyrode’s HEPES buffer as vehicle control or different concentrations of BML-111 (12.5, 25, and 50 µM) for 5 min at room temperature and then dispensed onto glass coverslips coated with fibrinogen (100 µg/ml) and incubated at 37 °C for 1 h. Samples were fixed with 0.2% (w/v) formyl saline and then permeabilised with 0.2% (v/v) TritonTM x-100. Adhered platelets were stained with Alexa-Fluor 488 Phalloidin for 1 h in the dark and mounted onto glass slides. Platelets were then visualised using confocal microscopy, objective (100x). Multiple images were taken randomly for each slide. (**a**) Representative image of platelet adhesion and spreading on fibrinogen. (**b**) An average number of platelets adhered. (**c**) Spreading of platelets was divided into 3 types: adhered, filopodia and lamelliopodia. (**d**) PRP was incubated with modified-Tyrode’s HEPES buffer or different concentrations of BML-111 (3.125, 6.25, 12.5, 25 and 50 µM) along with 10 µl red blood cells in glass test tubes for 15 min at room temperature. Thrombin (1 U/ml) was added to initiate clot formation. The clot was retracted around a sealed glass capillary tube placed in the middle of the tube. Clots were observed over 90 min. (**e**) The clot retraction was calculated by measuring the clot weights. The cumulative data were used to show clot weight. Data represent mean ± SEM (*n* = 3). *P ≤ 0.05, **P ≤ 0.01, ***P ≤ 0.001 and ****P ≤ 0.0001values were as calculated by One-way ANOVA.

Platelet spreading and clot retraction occur due to outside-in signaling events mediated by integrin αIIbβ3 following fibrinogen binding to ensure the stability of the growing thrombus. To assess the impact of BML-111 on the outside-in signaling, platelet spreading on fibrinogen coated coverslips was evaluated in BML-111 treated platelets. 25 and 50 µM BML-111 significantly reduced platelet adhesion treatment by 62% and 73%, respectively. Also, approximately 80% of platelets in vehicle-treated samples showed a high degree of lamelliopodia. On the other hand, pre-treatment with 25 and 50 µM of BML-111 inhibited the levels of platelet spreading to fibrinogen coated coverslips (Fig. 3a, b &c). Similarly, clot retraction was impeded by BML-111 treatment as shown in Fig. 3d &e. These findings indicate a role of FPR2/ALX in the formation and stability of thrombi.

### BML-111 negatively impacts calcium mobilisation

**Fig. 4:**
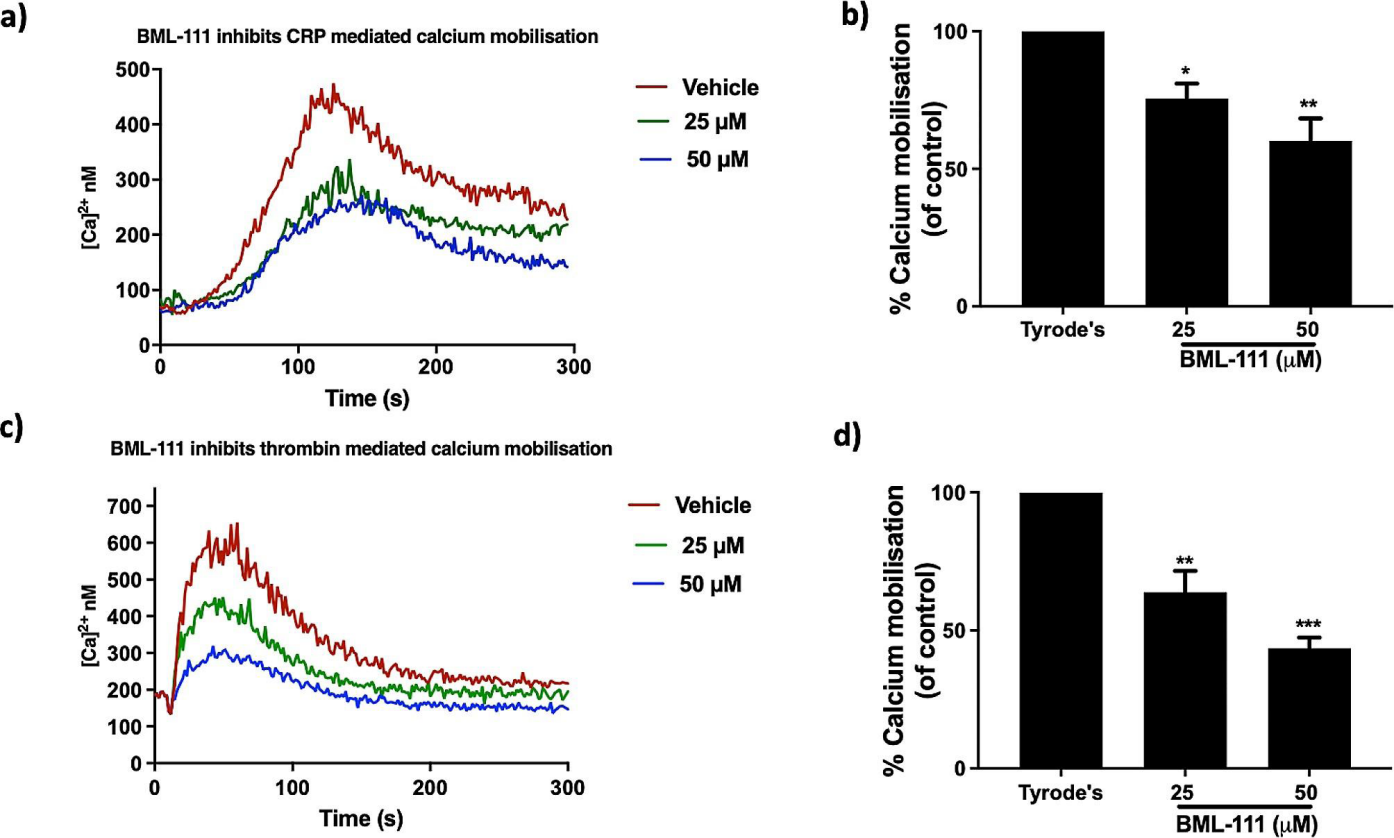
BML-111 inhibits intracellular Ca^2+^ mobilisation. Fura-2 AM-loaded platelets (4 × 10^8^ cells/ml) were treated with BML-111 (25 and 50 µM) or vehicle control (modified-Tyrode’s HEPES buffer) for 5 min, then stimulated with CRP-XL (0.25 µg/mL) or thrombin (0.05 U/mL) for 5 min. (**a**, **c**) Traces of cytosolic calcium mobilisation after stimulation with CRP-XL or thrombin. (**b**, **d**) Cumulative data of the peak intracellular Ca^2+^ levels were shown. Peak calcium levels achieved in the presence of vehicle control defines 100%. Fluorescent intensity was measured by a plate reader (with excitation at 340 and 380 nm and emission at 510 nm). Data represent the mean ± SEM (*n* = 4). *P ≤ 0.05, **P ≤ 0.01 and ***P ≤ 0.001 were as calculated by One-way ANOVA.

Knowing the important role of Ca^2+^ in several vital stages of platelet activation from inside-out signalling to degranulation, it is important to study the impact of BML-111 on intracellular Ca^2+^ mobilisation following CRP-XL or thrombin stimulation. A membrane permeable calcium dye, Fura-2AM was used to monitor the calcium levels through binding to free cytosolic Ca^2+^. As a result of Ca^2+^ binding, the dye gets excited at wavelength 340 and 380 nm and then emitted at 510 nm. Isolated platelets (4 × 10^8^ cells/ml) loaded with Fura-2AM were incubated with BML-111 (25 and 50 µM) or a vehicle control (modified-Tyrode’s HEPES buffer) for 5 min prior to stimulation with CRP-XL (0.25 µg/mL) or thrombin (0.05 U/mL) for 5 min. Levels of Ca^2+^ mobilisation were assessed by measuring the fluorescence. BML-111 treatment with 25 and 50 µM in CRP-XL stimulated platelets displayed an inhibition in Ca^2+^ levels as reflected in cytosolic Ca^2+^mobilisation. BML-111 at 25 and 50 µM showed a significant reduction in cytosolic calcium levels by approximately 25% and 40%, respectively, in comparison to the vehicle control (Fig. 4b). Similarly, a concentration-dependent reduction in calcium mobilisation was observed in 25 and 50 µM BML-111 treated platelets of 37% and 57%, respectively, in comparison to vehicle control following thrombin-stimulation (Fig. 4d).

### In vitro thrombus formation is reduced by BML-111

**Fig. 5:**
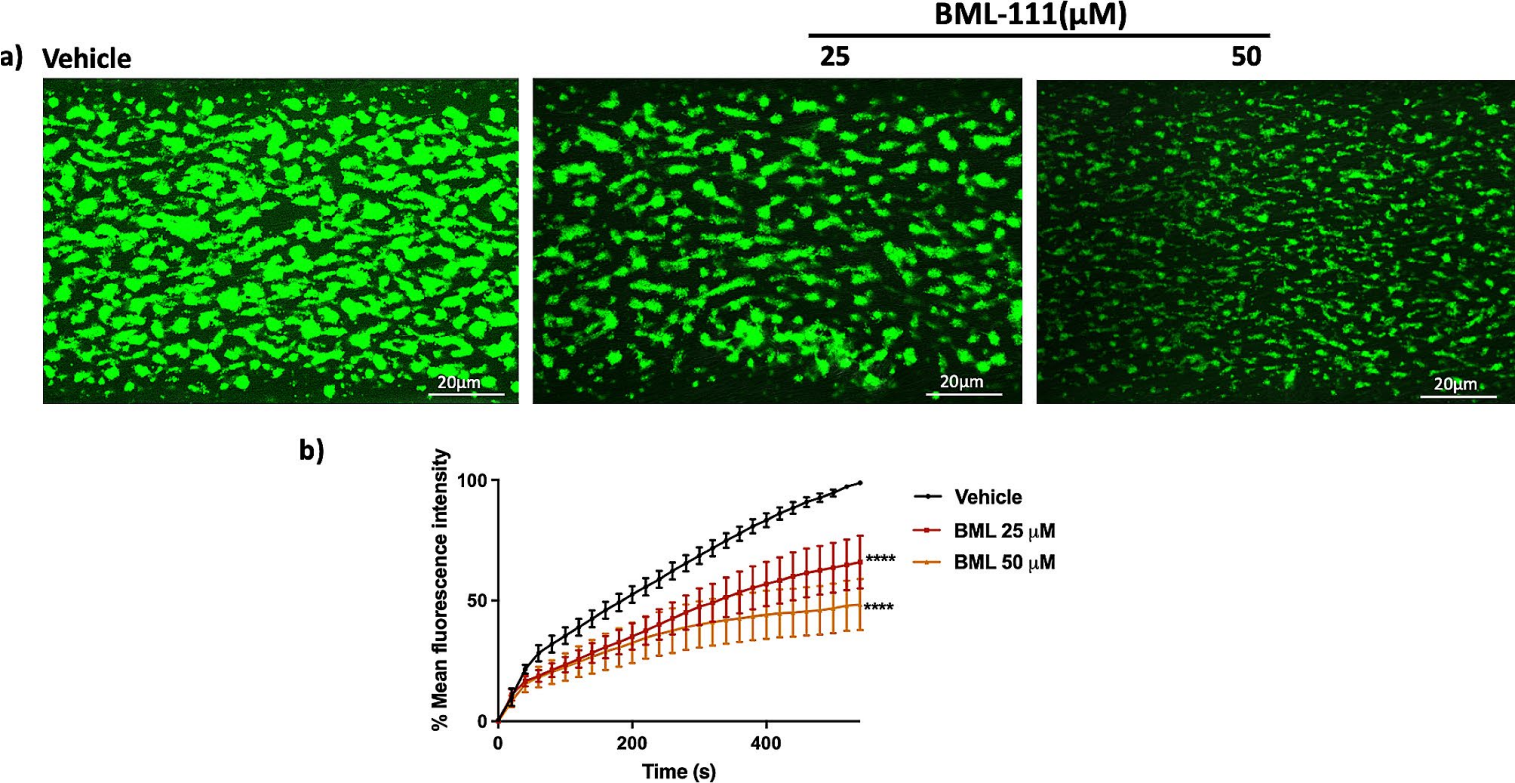
BML-111 attenuates thrombosis. A lipophilic dye, DiOC6 (5 µM) was added to citrated human blood and incubated for 1 h at 30 ℃, then treated with BML-111 (25 and 50 µM) or a vehicle-control (modified-Tyrode’s HEPES buffer) for 5 min. The labelled blood was then perfused across Vena8 Biochips, coated with collagen (100 µg/ml) under arterial flow conditions (shear stress: 20 dynes/cm^2^). (**a**) Representative images show thrombus formation over 540 s in 25 and 50 µM BMLL-111 or vehicle-control treated samples. Fluorescence was measured using an excitation wavelength of 488 nm and emission at 500–520 nm with an argon laser. A Nikon A1R confocal microscope (20x objective) was used to observe thrombus formation, and images were captured every second for over 600 s. (**b**) Quantified data represents mean thrombus fluorescence intensity for BML-111 and vehicle-control treated samples. The data were analysed using NIS-Elements software and normalised to the fluorescence level at the end of the assay in the vehicle-control treated sample. Data represent the mean ± SEM (*n* = 4), ****P ≤ 0.0001 was as calculated by Two-way ANOVA.

Given the impact of BML-111 on several aspects of platelet activation, the work was extended to assess the effect of BML-111 on thrombus formation in vitro under arterial flow conditions. Human whole blood was incubated with a lipophilic dye, DiOC6 (5 µM) at 30 °C for 1 h. Then, dye labelled whole blood was incubated with BML-111 or a vehicle-control for 5 min prior to perfusion through the collagen-coated chip at an arteriolar shear rate of 20 dynes/cm^2^. Fluorescence was excited at 488 nm with an argon laser and emission was measured at 500–520 nm. Thrombus formation on the microfluidic chip was observed by using a Nikon A1R confocal microscope with a 20X objective. Representative images at the end of the assay show big, stable, and bright formed thrombi in the vehicle-control treated sample (Fig. 5a). On the other hand, treatment with BML-111 significantly reduced the size of the formed thrombi. Collectively, 25, and 50 µM BML-111 treatment caused a significant reduction in thrombus formation by approximately 43% and 50%, respectively (Fig. 5b).

### BML-111 upregulates inhibitory pathway-mediated signaling

**Fig. 6:**
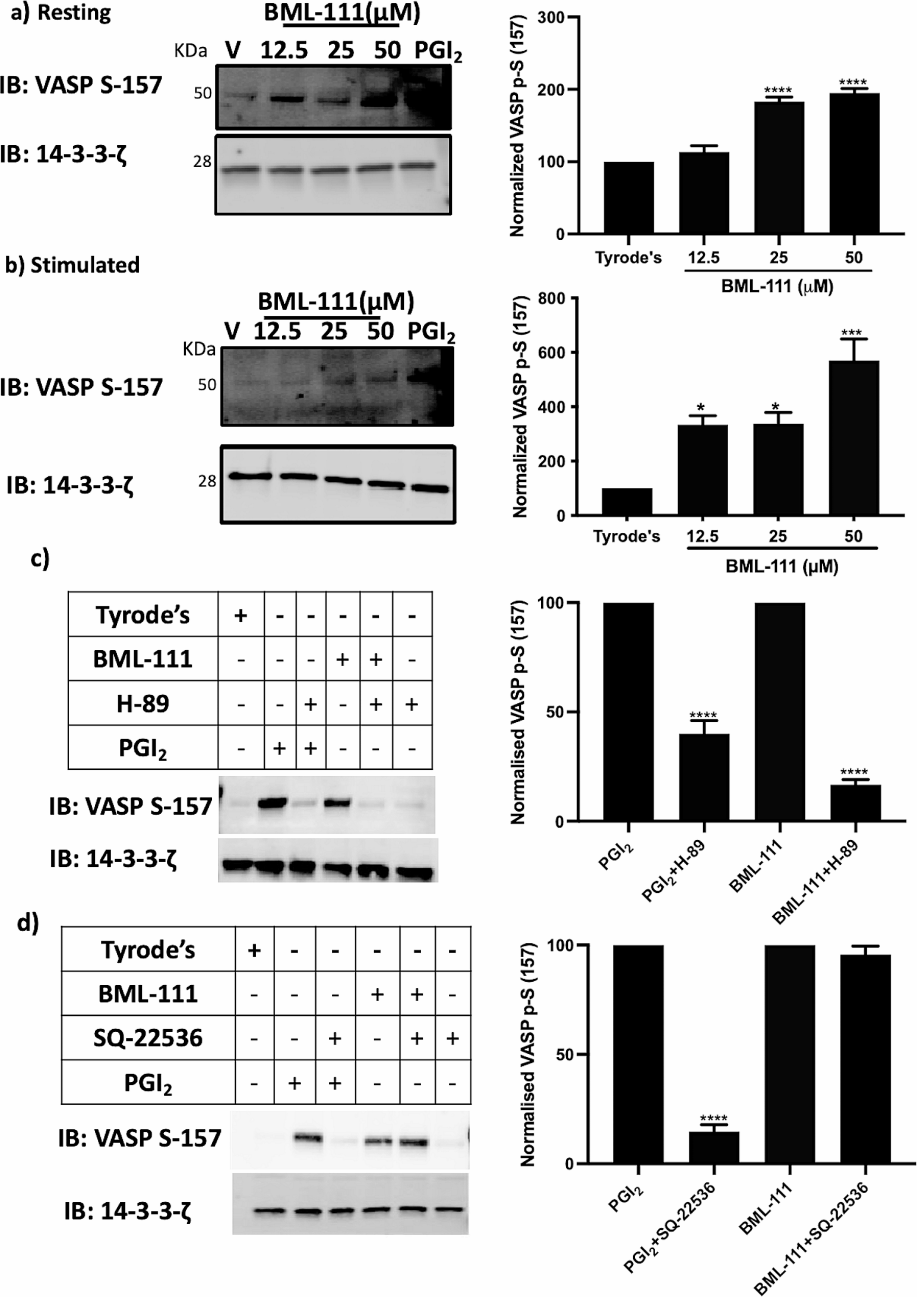
BML-111 regulates VASP S-157 phosphorylation via cAMP-independent PKA activation. Human isolated platelets (4 × 10^8^ cells/mL), under (**a**) Resting and (**b**) (0.1 U/ml) thrombin were pre-treated with BML-111 (12.5, 25 and 50 µM) or a vehicle-control (modified-Tyrode’s HEPES buffer) for 5 min then immunoblotted to detect VASP S-157 phosphorylation, a marker of PKA activity. Platelets were treated with PGI_2_ (1 µg/ml) as a positive control to stimulate the activity of PKA. Resting isolated human platelets (4 × 108 cells/mL) under resting conditions were treated with (**c**) H-89 (10 µM) or (**d**) SQ 22536 (100 µM) for 5 min before treatment with a vehicle-control (modified-Tyrode’s HEPES buffer) or BML-111(50 µM) for 5 min. Then, the samples were assayed for VASP S-157 phosphorylation. Treatment with PGI_2_ (1 µg/mL), which activates PKA by stimulating AC, was used as a positive control. Lysis of the samples was carried out using Laemmli sample buffer prior to separation by SDS-PAGE, then the samples were transferred to PVDF membranes. 14-3-3-ζ was detected by immunoblotting as a loading control. The impact of BML-111 on VASP S-157 phosphorylation is shown in representative blots from three distinct experiments. Data represent the mean ± SEM (*n* = 4). *P ≤ 0.05, ***P ≤ 0.001 and ****P ≤ 0.0001 were as calculated by One-way ANOVA

Gi-coupled FPR2/ALX has been linked to the regulation of platelet inhibitory signaling pathways of cAMP and hence increasing platelet reactivity. Nevertheless, BML-111 demonstrated a potential down-regulation of platelet activity and thrombosis. To further explain its inhibitory effect, we looked into the impact of BML-111 treatment on the phosphorylation of VASP-S157 and VASP-S239, markers of cAMP/PKA and cGMP/PKG activity, respectively, in resting and stimulated platelets. To achieve maximum phosphorylation levels of VASP S-157 and S-239, isolated platelets were treated with positive controls, PGI_2_ (1 µg/ml) and PAPA-Nonoate (100 µM; NO donor), respectively. As shown in Fig. 6a, pre-treatment of platelets with 25 and 50 µM BML-111 caused a significant increase in VASP S-157 phosphorylation by 86% and 88%, respectively, when compared to vehicle-treated platelets in resting conditions. Similarly, stimulated platelets showed an increase of VASP S-157 phosphorylation when treated with 12.5, 25 and 50 µM BML-111 in a dose dependent manner (Fig. 6b). However, when compared to vehicle samples, no changes have been noted in the phosphorylation levels of VASP S-239 in BML-111 treated samples in resting and stimulated conditions (supplementary Fig. 2a &b).

Given the effects of BML-111 on VASP S-157 phosphorylation, we assessed the levels of cAMP in the presence of BML-111. Interestingly, cAMP levels were not increased upon BML-111 treatment (supplementary Fig. 3). However, the elevation of cAMP levels was indeed observed in PGI_2_ treated control sample suggesting that BML-111 modulates VASP S-157 phosphorylation independently of cAMP. Moreover, H-89 dihydrocholride hydrate (10 µM), a selective inhibitor of PKA [14], and SQ 22536 (100 µM), an AC blocker which inhibits its activity and prevents cAMP production [15], inhibited VASP S-157 phosphorylation in the presence of PGI_2_ confirming that PGI_2_-induced VASP S-157 phosphorylation is modulated via cAMP/PKA signaling pathway. On the contrary, BML-111- mediated increase in VASP S-157 phosphorylation was completely abolished upon treatment with H-89. SQ 22536, on the other hand, failed to reverse BML-111 induced VASP S-157 phosphorylation (Fig. 6c &d). Together, these findings suggest that BML-111 exerts inhibitory effects towards platelet by activation of PKA via a cAMP-independent route.

## Discussion

Given the myeloid lineage of platelets and the wide range of receptors that platelets possess, a hypothesis was first raised by Czapiga et al. [8] that platelets express functional formyl peptide receptors, and these receptors are capable of inducing migration of platelets towards sources of formyl peptides. This was indeed proved valid as demonstrated by formyl peptide binding to platelets. Additionally, ligand binding to FPRs evoked calcium mobilisation and hence platelet activation [8, 13]. Such findings established a new role for FPRs in the regulation of platelet function in immunity and inflammation. However, the impact of FPR2/ALX and its anti-inflammatory ligand LXA4 in the regulation of platelet function and hence thrombosis were not determined.

LXA4, an eicosanoid that has been reported to exhibit a high binding affinity towards FPR2/ALX and a vital physiological role in the resolution of inflammation [3, 4, 16]. The fact that lipoxins in general have a short half-life and are prone to rapid enzymatic inactivation in vivo, may limit their efficacy in long experimental applications. Therefore, the need to design potent and more stable LXA4 analogues has strongly emerged [17]. 5(S), 6(R), 7-trihydroxyheptanoic acid methyl ester (BML-111), a C-7- truncated LXA4 analogue molecule, showed an inhibition of leukotriene B4-induced neutrophil chemotaxis equivalent to the levels exerted by LXA4 [17]. BML-111 since has been widely used as an LXA4 analogue to study the role of FPR2/ALX receptor. In the present study, we took a similar approach and used BML-111 to understand the role of this compound in platelet from a thrombotic research angle.

Using several independent techniques we established an increase in platelet binding of anti-FPR2/ALX antibody following platelet stimulation. Such upregulation in the receptor level is consistent with the reported increase in formyl peptide binding on human platelets upon thrombin stimulation [8]. Additionally, the study also showed an intracellular pool of the FPRs. We noted a similar observation when imaging FPR2/ALX in permeabilised and resting platelets with positive staining of the receptor across the cytosol, which was readily mobilised toward the platelet periphery following stimulation. Available evidence in the literature showed that neutrophils exhibit a similar mechanism of FPR translocation and upregulation upon cell activation [18, 19].

Given that BML-111 was not previously tested in platelets, it was prudent to test the selectivity of this compound for FPR2/ALX and to eliminate any chances of false-positive findings. The effect of BML-111 was tested in FPR2/ALX deficient platelets and BML-111 was found to be specific for the receptor as shown by the lack of effect of BML-111 treatment in the levels of agonist-mediated fibrinogen binding in FPR2/ALX deficient platelets. Available literature provides evidence for the role of FPR2/ALX as a modulator of platelet reactivity via ligation with antimicrobial peptides. Studies showed that endogenous anti-microbial peptide cathelicidin, LL3-7 primes platelets and induces thrombosis [9, 20]. However, Senchenkova et al. [21] reported that administration of FPR2/ALX pro-resolving and anti-inflammatory protein ligand AnxA1, attenuated platelet activation and thrombosis through inhibition of thrombin-mediated inside-out signaling and downregulation of integrins. Moreover, platelet activation and thrombus formation in the cerebral microvasculature were significantly suppressed following treatment with AnxA1 [22]. Similarly, the present study found that BML-111 has the ability to downregulate a range of platelet functions in vitro. Pre-treatment with BML-111 caused significant inhibition of platelet aggregation, degranulation and inside-out signaling. Similarly, clot retraction and platelet spreading were also affected. Such negative impact was also reflected in the reduced size of thrombi when whole blood treated with BML-111 was perfused over a collagen-coated surface in vitro. Also, PKA activity was increased upon FPR2/ALX stimulation with BML-111 independently of cAMP. The diversity of the signaling events and physiological responses upon stimulation of FPR2/ALX is quite a common feature exhibited by other GPCRs. These receptors do not follow a classic mode of ligand-receptor interaction where only a receptor is suggested to control the type of evoked signals and downstream effectors molecules, whereas, ligands would only determine the strength of generated signals. Instead, FPR2/ALX ligands have the ability to selectively activate certain downstream signaling pathways while inhibiting others, a phenomenon well known as biased agonism. In this concept, FPRs may bias toward either classical G proteins mediated signaling or another parallel pathway. The concept of biased agonism explains why different ligands such as LL-37 augment platelets activity while other pro-resolving ligands like AnxA1 and LXA4 analogue BML-111 exert inhibitory effects on platelets [23–27]. The reported priming effects of FPR2/ALX anti-microbial peptide, LL-37 on platelets [9, 20] follow a classical GPCR signaling pathway in which ligated FPR2/ALX functions via Gi-coupled signaling pathway, which reduces cAMP levels. The cAMP has an inhibitory impact on platelets, and its suppression is responsible for the aforementioned platelet priming and activation. Whereas, BML-111 induced attenuation of platelet activation is likely due to the activation of another effector pathway downstream of FPR2/ALX. In conclusion, the present study uncovers for the first time the biased agonism behaviour of FPR2/ALX receptor via BML-111 ligand in platelets and the protective potentials of BML-111 against thrombosis through non classical GPCR mode.

## Electronic supplementary material

Below is the link to the electronic supplementary material.

Supplementary Material 1

## Data Availability

No datasets were generated or analysed during the current study.
