# Editorial expression of concern: Lipoxin A4 analogue, BML-111, reduces platelet activation and protects from thrombosis

**DOI:** 10.1186/s12959-024-00679-4

**Published:** 2024-12-05

**Authors:** Shatha AlOmar, Joanne L. Mitchell, Eman AlZahrani

**Affiliations:** 1 Department of Clinical Laboratory Sciences, King Saud University, Prince Turki Ibn Abdulaziz Al Awwal Rd, Riyadh, 12371 Saudi Arabia; 2 Cardiovascular Sciences, University of Birmingham, Birmingham, UK; 3 School of Pharmacy, University of Reading, Reading, UK

The Editors are issuing this editorial expression of concern to inform the authors that concerns were raised regarding the incorrect data availability statement present in this article [1]. The authors were contacted with concerns and for providing raw data for this article, however, despite several efforts, the Publisher is unable to get any response from the authors.

The readers are therefore requested to interpret the data presented in this article with caution.

None of the authors respond to correspondence from the Publisher about this Editorial Express of Concern.
