# Supplementary material for: Lipoxin A4 analogue, BML-111, reduces platelet activation and protects from thrombosis
Source: Thromb J. 2024 Apr 23;22:39. doi: 10.1186/s12959-024-00606-7 (PMC11036777; doi:10.1186/s12959-024-00606-7)
Supplement: Supplementary file 1 — Supplementary Material 1 [file 12959_2024_606_MOESM1_ESM.docx]

**Lipoxin A4 analogue, BML-111, reduces platelet activation and protects from thrombosis**

Shatha AlOmar^1,3^, Joanne L Mitchell^2^ and Eman AlZahrani^3^

^1^ Department of Clinical Laboratory Sciences, King Saud University, Riyadh, KSA

^2^ Cardiovascular Sciences, University of Birmingham, Birmingham, UK

^3^ School of Pharmacy, University of Reading, Reading, UK

**Materials**

BML-111, H-89 and SQ-22536 inhibitors were purchased from Bio-Techne Ltd. (Abingdon, UK). Professor Richard Farndale (University of Cambridge, UK) supplied the cross-linked collagen related peptide (CRP-XL). GPRP (Gly-Pro-Arg-Pro) peptide, cangrelor, indomethacin, human thrombin, U46619, EGTA (Ethylene glycol-bis(b-aminoethyl ether)- N,N,N’,N’-tetraacetic acid), fibrinogen from human plasma and 3,3′-dihexyloxacarbocyanine iodide (DiOC6; 98%) were purchased from Sigma–Aldrich (Poole, UK). MRS2179 was purchased from Abcam (Cambridge, UK). Bovine serum albumin (BSA) and Fura-2 AM were from Thermo-Fisher Scientific (Loughborough, UK). Phosphate-Buffered Saline (PBS) tablets and Alexa Fluor® 488 labelled phalloidin were from Life Technologies (Paisley, UK). 2- Morpholinoethanesulfonic acid hydrate (MES) was purchased from Fluorochem (Hadfield, UK) The poly-l-lysine coated-12mm coverslips were from VWR International Ltd (Lutterworth, UK). 10% Mini-PROTEAN® TGX™ precast polyacrylamide gels, extra thick blot papers and dual-stained molecular weight markers were obtained from Bio-Rad (Hemel Hempsted, UK). Vena8 Fluoro+TM Biochips were purchased from Cellix OEM Microfluidic Solutions (Dublin, Ireland). The polyvinylidene difluoride (PVDF) blotting membrane was obtained from GE Healthcare, UK. A cAMP ELISA kit was purchased from Cayman Chemical (Cambridge, UK). All other reagents used were at analytical grade purchased from SigmaAldrich (Poole, UK). *Fpr 2/3 ^-/-^* on C57BL/6 background mice (platelet specific conditional knock out mice) were obtained from Prof. Maurio Perretti from William Harvey Research Institute, London and the colony was established at the University of Reading. The wild type control mice (Cx57BL/6) were purchased from Envigo (Huntingdon, UK).

**Methods**

Human blood was taken from consenting, drug-free volunteers on the day of the experiment according to the methodology approved by the University of Reading Research Ethics Committee. Blood was taken using 3.8% (w/v) sodium citrate and Acid Citrate Dextrose (ACD; 110 mmol/L glucose, 80 mmol/L citric acid, 120 mmol/L sodium citrate) as an anticoagulant. Whole blood was centrifuged at 102g for 20 minutes at 20°C to yield platelet-rich plasma (PRP). Where washed platelets were required, they were isolated from the PRP by further centrifugation at 1413g for 10 minutes at 20°C in the presence of 0.1 μg/ml prostacyclin to prevent activation. The supernatant was discarded in Klorsept disinfectant (Medentech, Wexford, Ireland) and the platelet pellet was resuspended in 25ml of modified Tyrodes-HEPES buffer (134 mmol/L NaCl, 0.34 mmol/L Na2HPO4, 2.9 mmol/L KCl, 12 mmol/L NaHCO3, 20 mmol/L HEPES, 5 mmol/L glucose, 1 mmol/L MgCl2, pH 7.3) and 3 ml of ACD in the presence of 0.1 μg/ml prostacyclin. Platelets were centrifuged at 1413g for 10 minutes at 20oC and resuspended to a density of 4x108 cells/ml in modified Tyrodes-HEPES buffer using a platelet count obtained with a Z Series Coulter Counter (Beckman Coulter, CA, USA). Washed platelets were rested for at least 30 minutes at 30 °C prior to the experiment to allow responses to recover. Platelet preparations typically contained fewer than 1 contaminating erythrocyte or leukocyte per 6500 platelets.

**Western blotting**

Human washed platelets were prepared at a density of 8x10^8^ cells/ml and lysed by adding 6X Laemmli sample reducing buffer [4% (w/v) SDS, 20% (v/v) glycerol, 0.5M Tris, 0.001% (w/v) Brilliant Blue R and 10% (v/v) 2-mercaptoethanol]. To study cell signalling, human washed platelets were prepared at a density of 4x10^8^ cells/ml under non-aggregation conditions using indomethacin (20 µM), cangrelor (1 µM), MRS2179 (100 µM) and EGTA (1 mM). Platelets were then treated with BML-111 or vehicle control (modified-Tyrode’s HEPES buffer) for 5 minutes and then stimulated with platelet agonists in the aggregometer. Resting or stimulated samples were lysed with 6X Laemmli sample reducing buffer and heated to 95 ^o^C for 5 minutes prior to storing at -20 ^o^C until use. Proteins were separated by SDS-PAGE using 4-20% Mini-PROTEAN TGX precast protein gels. Samples were heated to 95^o^C for 5 minutes again before loading into gels submerged in 1X Tris/Glycine/SDS buffer (25 mM Tris, 192 mM glycine, 0.1% SDS, pH 8.3) within a Mini-PROTEAN tetra vertical electrophoresis cell (Bio-Rad, CA, USA). Electrophoresis was run for 1 hour at a constant voltage of 150V. Separated proteins were transferred to a polyvinylidene difluoride (PVDF) membrane using semi-dry western blotting (Trans-Blot SD Semi-Dry Transfer Cell; BioRad, CA, USA). PVDF membrane soaked in methanol was placed below the resolving gel in the transfer cell and sandwiched between 4 sheets of 3MM filter paper soaked in cathode buffer (25 mM Tris-base, 40 mM 6-amino-N-hexanoic acid; pH 9.4) placed at the top and 4 sheets of 3MM filter paper soaked in anode buffer (300 mM Tris-base, 20% (v/v) methanol; pH 10.4) placed at the bottom. A constant voltage of 15V was applied to this setup for 2 hours to facilitate efficient transfer of proteins from gel to membrane. PVDF membranes were transferred into a 5% (w/v) solution of bovine serum albumin (BSA) dissolved in Tris-buffered saline with Tween 20 (TBS-T) (20 mM Tris, 140 mM NaCl, 0.1% Tween, pH 7.6) to block the membrane for 1 hour at room temperature. Primary antibodies were added into a 2% (w/v) solution of BSA (dissolved in TBS-T) and membranes were incubated with these solutions overnight at 4 ^o^C on a rotator. Primary antibody solutions were removed from the PVDF membranes the next day and membranes were washed three times for 10 minutes each with TBS-T. Secondary antibodies were added to a 2% (w/v) BSA (dissolved in TBS-T) solution, which was then added to PVDF membranes and incubated in the dark at room temperature for 1 hour. PVDF membranes were washed three times again for 5 minutes each with TBS-T. PVDF membranes were scanned using a Typhoon FLA 9500 (Amersham Biosciences, Buckinghamshire, UK), and quantification of the fluorescence intensity of individual bands was determined using Image Quant software version 8.1 (GE Healthcare).

**Flow cytometry analysis**

Flow cytometry-based assays were used to detect P-selectin exposure and fibrinogen binding on the platelet surface as a measure for platelet α-granule secretion and integrin αIIbβ3 activation, respectively. These assays were employed in this study to analyse platelet activation. 5 μl of PRP were incubated with 1μl of rabbit anti-human fibrinogen antibodies labelled with fluorescein isothiocyanate (FITC) (Dako, Uk), and phycoerythrin-Cy5 (PE-Cy5)-conjugated mouse anti-human CD62P (P-selectin) antibodies (BD Biosciences, UK), and 44.5 μl of HEPES-buffered solution to make up the final volume of 50 μl in presence of BML-111 or a vehicle-control. Platelets were stimulated using 0.5 μg/ml CRP-XL, 0.1 U/ml thrombin 25 μg/ml of Gly-Pro-Arg-Pro (GPRP) was added to prevent fibrin polymerization] for 20 minutes at RT, and the reaction was stopped by adding 0.2% (v/v) formyl saline. Appropriate isotype controls or EGTA were used to obtain negative controls for the antibody responses. Flow cytometric acquisition was performed using a BD Accuri C6 flow cytometer (BD Biosciences, UK), and data collected from 10,000 gated events for platelets were analysed using the BD Accuri C6 software. To examine the level of fibrinogen binding and P-selectin exposure, the median fluorescence intensity was calculated.

**Immunocytochemistry**

PRP was prepared as described earlier. Platelets were treated with an agonist (5 μM U46619, a thromboxane A2 analogue, this would mediate platelet activation with minimal changes in platelet shape allowing better visualisation of FPR2/ALX distribution ) or a vehicle control under stirring conditions at 37˚C for three minutes using an aggregometer prior to fixation and centrifugation. Platelet pellet was then re-suspended in 500 μl of modified Tyrode’s-HEPES buffer and centrifuged at 1000 g for 10 minutes. Final platelet pellet was then re-suspended in 500 μl modified Tyrode’s HEPES buffer containing 1% (w/v) BSA. Poly-l-lysine coated-12 mm glass coverslips were placed in 6x4 well cell culture plates. 90 μl of platelets were dispensed onto each coverslip and left for 60 minutes at 37˚C to allow them to adhere to the glass surface. Coverslips were washed three times for one minute each using PBS to remove non-adherent platelets, followed by blocking using 1% (w/v) BSA for 60 minutes at room temperature. Fixed platelets were permeabilised with 0.2% (v/v) TritonTM X-100 and then incubated with the primary antibody for overnight at 4˚C. Stained coverslips were washed three times for one minute each using PBS to remove unbound primary antibodies, and secondary antibodies were added and left for incubating in the dark for 60 minutes at room temperature. Platelets were then washed three times for one minute each using PBS followed by mounting on glass slides using a mounting media (Life Technologies, UK). Immunofluorescence was analysed by confocal microscopy using an oil immersion objective (100x) and filters corresponding to the type of fluorophores applied to detect the staining.

**Platelet aggregation assay**

Light transmission aggregometry (LTA) using an optical aggregometer was used to monitor the agonist-induced changes in turbidity/aggregation of platelet suspension with constant stirring (1,200rpm) at the physiological temperature (37 °C). We monitored the effects of different concentrations of BML-111 on platelet activation using isolated platelets in a buffered solution to understand its direct effects on platelets. Human isolated platelets (245 µl) at 4x10^8^ cells/ml were treated with either a vehicle control [0.1 % (v/v) Modified-Tyrode’s HEPES buffer] or a range of different concentrations of BML-111 (3.125, 6.25, 12.5, 25, and 50 µM) for five minutes prior to the addition of 50 µl of an agonist (CRP-XL or thrombin). Platelet aggregation was recorded for five minutes using an optical aggregometer (Chrono-log, USA). The percentage of aggregation obtained at 300 seconds was calculated and compared with the control samples to estimate the level of inhibition.

**Dense granule secretion**

Dense granule secretion was analysed by measuring the release of ATP from platelets. The ATP release was monitored concurrently, with aggregation in a Lumi- aggregometer (model 700) by using luciferin-luciferase kit (Chrono-Log Corporation, Havertown, PA, USA). A volume of 237 µl of PRP was treated with 3 µl of different concentrations of BML-111 (3.125, 6.25, 12.5, 25, and 50 µM) at 37°C for five minutes and the incubation was performed under non-stirring condition. Luciferin-luciferase reagent (30 μl) was then added under nonstirring conditions and incubated for 2 minutes. 30 μl agonist (CRP-XL or thrombin) was then used to simulate the platelets under stirring conditions. The ATP release and aggregation traces were recorded for five minutes at 37°C.

**Intracellular Ca^2+^ mobilisation measurement**

The mobilisation of intracellular Ca^2+^ from intracellular stores into platelet cytosol was evaluated in a fluorescence-based 96-well plate assay by using a dual excitation calcium sensitive fluorescent dye Fura-2 AM. Human PRP was incubated with Fura-2 AM (2 μM) for 1 hour at 30 °C then followed by centrifugation at 350 g for 20 minutes. The platelet pellet was resuspended in modified Tyrode’s-HEPES buffer at (4 × 10^8^ cell/mL). Then, Fura-2- loaded isolated platelets were incubated with BML-111 or a vehicle-control for 5 minutes at 37 °C prior to stimulation with CRP-XL or thrombin. Ca^2+^ mobilisation was measured in the presence of EGTA (10 mM), to chelate extracellular Ca^2+^, thereby allowing the measurement of only Ca^2+^ store release efflux. Fluorescence measurements (at excitation 340 nm and 380 nm, and emission at 510 nm) were taken for 5 minutes using a plate reader. Data were analysed by calculating the percentage of calcium released at 90 seconds.

**Platelet spreading**

Human isolated platelets at a density of 2x10^8^ cells/ml were prepared and left to rest for 30 min at 30°C. Glass coverslips were placed in a 4 x 3 well culture plate and coated with fibrinogen (100μg/ml) for 1 hr at room temperature. Then they were blocked with 1% (w/v) BSA for 1 hr at room temperature. Coverslips were washed 3 times for 1 min each with PBS. Then, 300 μl of platelets (pre-incubated for 5 min with 0.1 % (v/v) modified-Tyrode’s HEPES buffer or different concentrations of BML-111) were dispensed onto each coverslip and incubated for 1 hr at 37°C to allow platelet adherence and spreading. Coverslips were washed 3 times for 1 min each with PBS to remove non adherent platelets and fixed with 0.2% formyl saline for 10 min at room temperature followed by washing three times with PBS. Fixed platelets were permeabilised with 0.2% (v/v) TritonTM x-100 and incubated with Phalloidin for 1 hr in the dark at room temperature. Stained coverslips were then washed three times for one minute each using PBS followed by mounting on glass slides using a mounting media (Life Technologies, UK). Immunofluorescence was analysed by confocal microscopy using an oil immersion objective (100x) and filters corresponding to the type of fluorophores applied to detect the staining.

**Clot retraction assay**

PRP was prepared and left to rest for 30 min at 30°C. 10 μl of red blood cells and 5 μl of either a vehicle control 0.1 % (v/v) modified-Tyrode’s HEPES buffer or BML-111 at a range of different concentrations (3.125, 6.25, 12.5, 25, and 50 µM) were added to the human PRP (200 μl) in test tubes. The final volume was made up to 1ml by adding 0.1 % (v/v) modified Tyrode’s HEPES buffer (containing CaCl_2_) and the mixture was left to incubate for 15 min at room temperature. Then, 10 μl human thrombin (final concentration 1 U/ml) was added to generate the clot. A blunted glass capillary tubes were added in the centre of the test tubes to establish a surface for the contraction and generation of the clots. Clots were recorded at 30 minute intervals for up to 90 minutes by taking photos. The generated clots were weighted by using a microbalance at the end of the assay to calculate the rate.

***In Vitro* thrombus formation under arterial flow conditions**

Human whole blood was incubated with a lipophilic dye, DiOC6 (5 µM) at 30 °C for 1 hour. Vena8 BioChip microfluidic channels were coated with type I collagen (100 μg/mL) for 1 hour and excess collagen was washed with modified Tyrode’s-HEPES buffer. Dye labelled whole blood was incubated with BML-111 or a vehicle-control for 5 minutes prior to perfusion through the collagen-coated chip at an arteriolar shear rate of 20 dynes/cm^2^. Fluorescence was excited at 488 nm with an argon laser and emission was measured at 500– 520 nm. Thrombus formation on the microfluidic chip was observed by using a Nikon A1R confocal microscope with a 20X objective. Images of single sections were captured every second for 540 seconds. The mean thrombus fluorescence intensity was calculated using NIS-Elements software (Nikon, Tokyo, Japan).

**cAMP ELISA assay**

A cAMP immunoassay kit based on a competitive ELISA principle was used to measure cAMP levels in platelets, according to the protocol provided by the manufacturer (Cayman chemical, Cambridge, UK). 250 μL human isolated platelets (4 × 10^8^ cells/mL) were incubated with (5 μL) BML-111 (12.5, 25, and 50 µM) or a vehicle-control for 5 minutes. Then, the samples were stimulated with 1 μg/ml CRP-XL or 0.1 U/ml thrombin. After 5 minutes of stimulation, a lysis buffer (0.1 M HCL) was added to the samples. Then, samples were immediately placed in -20°C. The samples were later thawed and added to microwells pre-coated with mouse monoclonal anti-rabbit IgG. The association between cAMP concentrations and absorbance was determined using cAMP standards (serial dilutions, 50 μL). Then, the assay plate was covered with plastic film and incubated for 18 hours at 4°C. After incubation, the wells were emptied from the contents and were washed with washing buffer five times. Then, Ellman’s Reagent (200 μL) was added to the wells and the plate was incubated in the dark on a horizontal orbital plate shaker for 90-120 minutes at room temperature. The absorbance was measured at a wavelength between 405 and 420 with a plate reader. A standard curve was plotted from the absorbance readings of the cAMP standards.

## Lactate dehydrogenase (LDH) cytotoxicity assay

Cell necrosis (death) occurs due to external factors such as injuries, trauma and chemotherapy. Necrotic cells release an enzyme, lactate dehydrogenase and this can be measured as a marker for cytotoxicity when pharmacological agents are tested. Here, to determine whether BML-111 exerts any cytotoxic effects on platelets, LDH release was measured using an LDH Cytotoxicity Assay Kit (ThermoScientific, UK). PRP was treated with either a vehicle control [0.1 % (v/v) Modified-Tyrode’s HEPES buffer] or a range of different concentrations of BML-111 (3.125, 6.25, 12.5, 25, and 50 µM) for five minutes at 37°C in 5% CO_2_. Following incubation 25μl of treated samples were transferred to a 96-well plate and mixed with 25μl of LDH reaction mix (provided with the kit) and incubated at room temperature for 30 minutes. The reaction was stopped by the addition of 25μl of stop solution (provided with the kit) and the absorbance of the reaction mix was measured at 680 nm and subtracted from the absorbance obtained at 490 nm to eliminate the background noise. The resulting LDH activity was normalised using an equation as recommended by the manufacturer: % cytotoxicity = [compound -treated LDH activity – spontaneous LDH activity/ maximum LDH activity – spontaneous activity] *100.

**Statistical analysis**

The statistical significance for studies with more than 2 groups comparison was assessed using one-way analysis of variance (ANOVA). The *in vitro* thrombus formation assay was analysed using two-way ANOVA. Data are presented as mean ±SEM and a P-value of ≤ 0.05 was considered to be statistically significant. Statistical analysis was performed using GraphPad Prism software (GraphPad, San Diego, CA, version 8.00).

**Supplementary figures.**


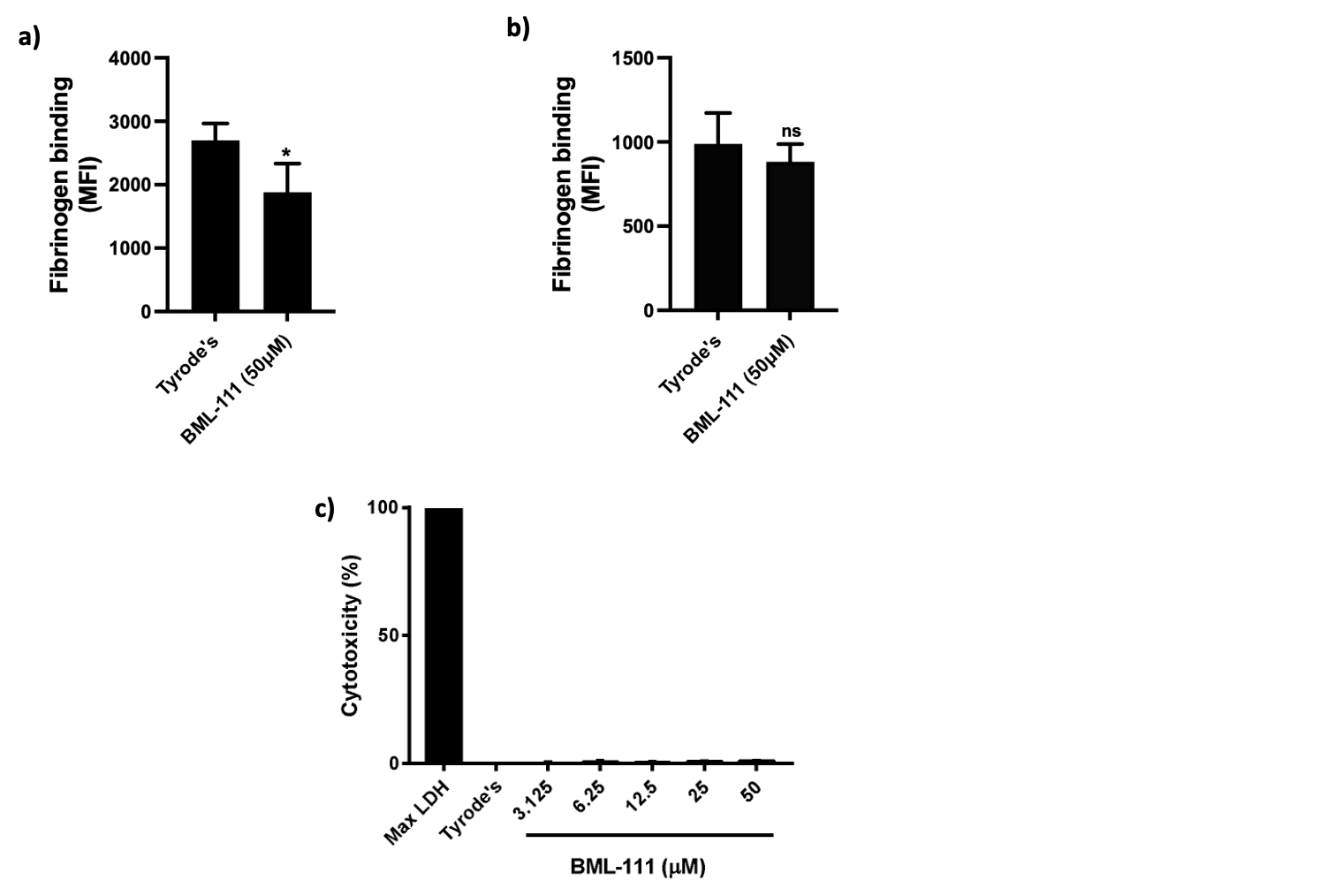


**Suppl. Figure 1: BML-111 selectively binds FPR2/ALX in platelets.** Mouse PRP from (a) wild type (FPR2/ALX+/+) and (b) FPR2/ALX-/-deficient mice were incubated with BML-111 (50 µM) and vehicle containing control in the presence of FITC-conjugated anti-human fibrinogen antibody for 5 minutes. CRP-XL (0.5 µg/ml) was used to stimulate the reactions for 20 minutes. The samples were then fixed with 0.2% (v/v) formyl saline and examined by a flow cytometer. Data represent the mean of median fluorescence intensity ± SEM (n = 6). *P ≤ 0.05 value was as calculated by One-way ANOVA. ns: not significant. (c) Human isolated platelets were treated with a positive control, a vehicle control, or various concentrations of BML-111 (3.125-50 μM), for 30 minutes and the release of LDH, a marker for cytotoxicity was measured at 490 and 650 nm using spectrofluorometer. The LDH release attained with the positive control was considered as 100%, and the levels of LDH release for BML-111-treated samples were calculated. Data represent mean ± SEM. (n = 3). Statistical significance was analysed by one-way ANOVA.

**
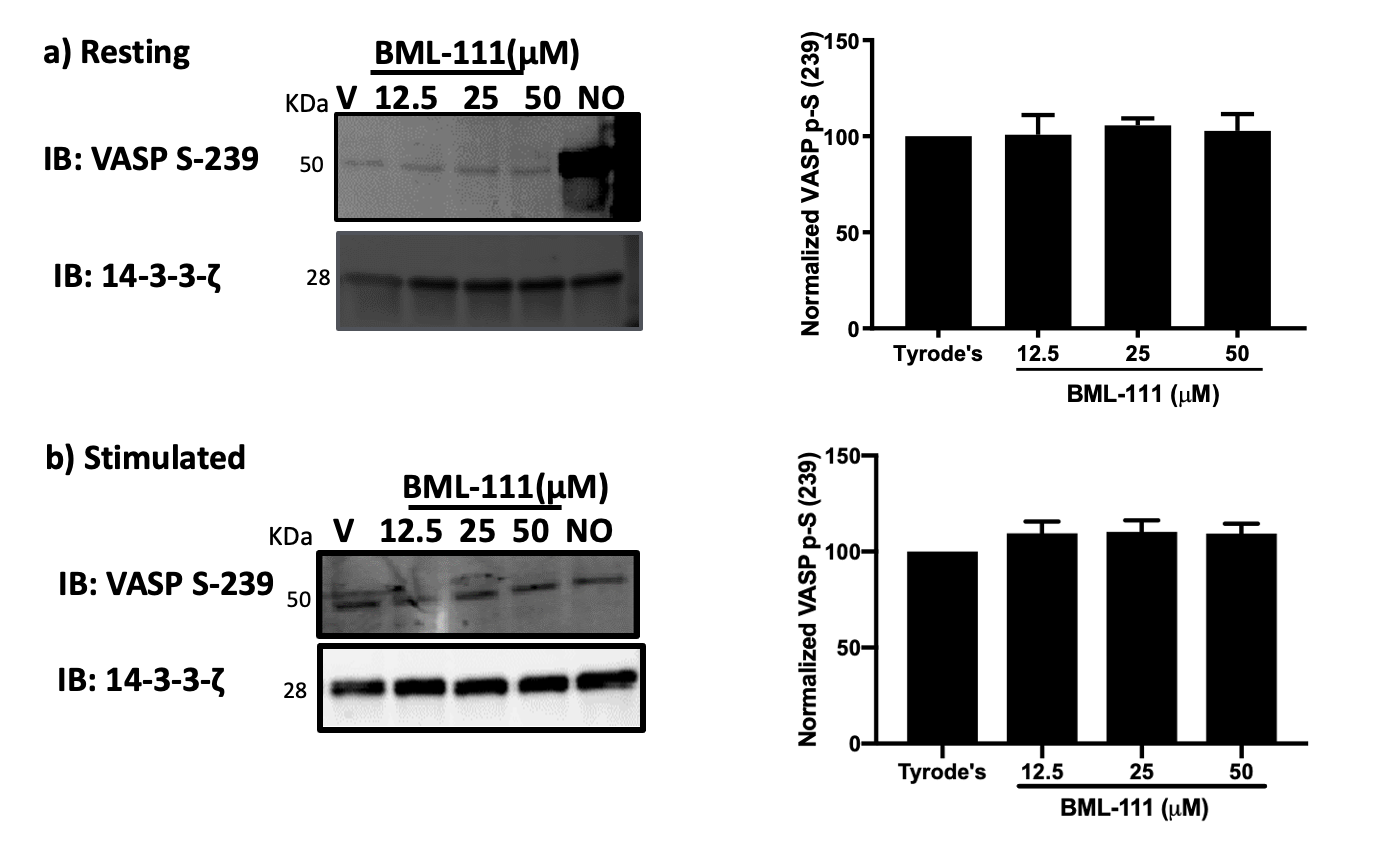
**

**Suppl. Figure 2:** **BML-111 does not modulate PKG activity.** Human isolated platelets (4 × 10^8^ cells/mL), under (a) Resting and (b) (0.1 U/ml) thrombin were pre-treated with BML-111 (12.5, 25 and 50 μM) or a vehicle-control (modified-Tyrode’s HEPES buffer) for 5 minutes then immunoblotted to detect VASP S-239 phosphorylation, a marker of PKG activity. Platelets were treated with PAPA-Nonoate (100 μM) as a positive control to stimulate the activity of PKG. Laemmli sample buffer was used to lyse the samples before separation by SDS-PAGE and transfer to PVDF membranes. 14-3-3-ζ was applied as a loading control. Data represent the mean ± SEM (n = 4).


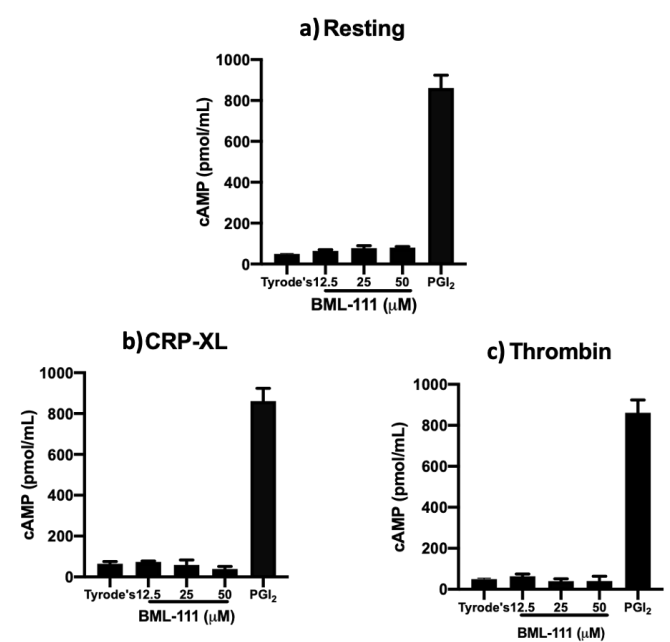


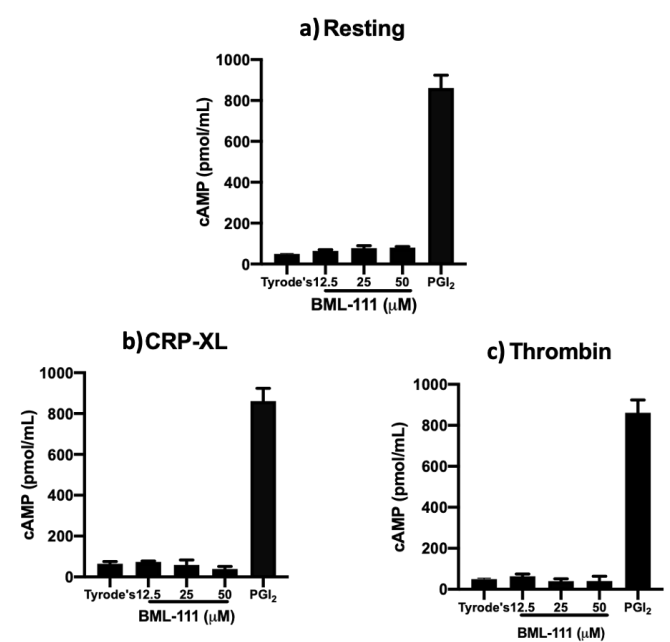

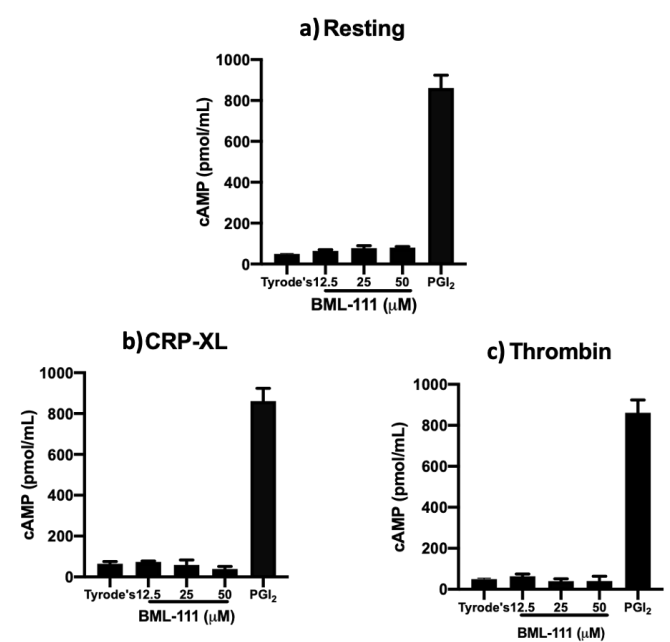


**Suppl. Figure 3:** **BML-111 does not impact cAMP levels in platelets**. Resting and stimulated isolated human platelets (4 × 108 cells/mL) were treated with BML-111 (12.5, 25 and 50 μM) or a vehicle-control (modified-Tyrode’s HEPES buffer) for 5 minutes. (a) unstimulated, (b) 1 μg/ml CRP-XL and (d) 0.1 U/ml thrombin stimulated platelets for 5 minutes at 37 o C in an aggregometer. PGI2 (1 μg/mL) was used as a positive control. HCl was used to stop the reactions. A cAMP ELISA kit was used to determine the concentrations of cAMP according to the manufacture protocol. The cAMP concentrations (pg/mL) produced by platelets incubated with BML-111 or PGI2 in (a) resting or stimulated platelets with (b) CRP-XL or (c) thrombin are presented. Data represent the mean ± SEM (n = 3).
